# Improved Mitigation of Cyber Threats in IIoT for Smart Cities: A New-Era Approach and Scheme

**DOI:** 10.3390/s21061976

**Published:** 2021-03-11

**Authors:** Semi Park, Kyungho Lee

**Affiliations:** School of Cybersecurity, Korea University, Seoul 02841, Korea; semi0502@korea.ac.kr

**Keywords:** anomaly detection, data envelopment analysis, smart city, industrial control systems, cybersecurity

## Abstract

Cybersecurity in Industrial Internet of Things (IIoT) has become critical as smart cities are becoming increasingly linked to industrial control systems (ICSs) used in critical infrastructure. Consequently, data-driven security systems for analyzing massive amounts of data generated by smart cities have become essential. A representative method for analyzing large-scale data is the game bot detection approach used in massively multiplayer online role-playing games. We reviewed the literature on bot detection methods to extend the anomaly detection approaches used in bot detection schemes to IIoT fields. Finally, we proposed a process wherein the data envelopment analysis (DEA) model was applied to identify features for efficiently detecting anomalous behavior in smart cities. Experimental results using random forest show that our extracted features based on a game bot can achieve an average F1-score of 0.99903 using 10-fold validation. We confirmed the applicability of the analyzed game-industry methodology to other fields and trained a random forest on the high-efficiency features identified by applying a DEA, obtaining an F1-score of 0.997 using the validation set approach. In this study, an anomaly detection method for analyzing massive smart city data based on a game industry methodology was presented and applied to the ICS dataset.

## 1. Introduction

A smart city is an urban model envisioned to solve urban problems and improve the quality of life of residents by integrating technologies such as information and communications technology (ICT) and big data. However, there is no clear and common definition of a smart city because each country and institution defines the term from its own perspective [1,2]. Hence, there are various definitions of smart cities. However, they commonly aim to solve urban problems and improve the quality of life for residents through technology. The concept of a smart city emerges against highly diverse backdrops; therefore, the unified conceptualization of what it entails was achieved relatively recently. The origin of smart cities can be divided into urban planning and global crisis response aspects. Smart cities were initially understood as goals to be attained in terms of advanced automation and networking. However, they are often defined as integrated heterogeneous platforms. A smart city is primarily envisioned to improve the quality of life of residents and regional competitiveness using ICT networks.

A smart city is defined as the infrastructure itself. It provides services such as transportation, hydropower, smart health, and a smart grid through its connections to existing conventional infrastructure [3,4]. IIoT is an integral part of the smart city that helps constitute the critical infrastructures [5]. Yet, the ever increasing cyber attacks have emphasized the significance of the safety and security of IIoT in smart cities [6]. The campaign behind UNC2452 has been accounted for trojanizing the victims through SolarWind’s Orion IT monitoring and management software [7]. The incident is estimated to have taken place in early 2020, and the victims have included the government agencies, multi-conglomerate organizations from North America, Europe, Asia, and the Middle East [8]. Cyber-attack weapons and cyber talents are the core of cyber-attacks that pose a serious threat to a country’s cyber security. As such, technologies regarding the cyber threat detection are being developed. In order to better protect the sensitive information produced from the smart health domain, a data-driven approach towards security is required to detect the various anomalies [9,10].

Smart cities create massive amounts of data because they connect various heterogeneous devices [11]. To analyze such large-scale data, intelligent anomaly detection techniques using machine learning (ML) have been studied [12]. Similar to the smart city field, the game design field generates large-scale data and is based on data analysis techniques. Furthermore, both smart cities and online game worlds aim to provide a level of user satisfaction. Online games are a representative commercially developed field; a massive number of users access and play these games in real time. Companies developing such games have grown in proportion to the number of users accessing them; thus, user turnover results in a loss of revenue. It is therefore essential to detect anomalies that cause such turnover [13]. To detect abnormal behavior in big data in the game field, data analysis research using ML and deep learning is being conducted. The game security methodology used in existing research has revealed abnormal behavior with a high level of accuracy. Smart cities need to analyze large amounts of data generated by large numbers of users with high accuracy. Therefore, we can adapt similar approaches to game methodologies.

This study is an attempt to detect anomalies using the methodology used in the field of game security. In addition, considering the diversity of smart cities, we propose a process that can identify an efficient feature set for anomaly detection through a data envelopment analysis (DEA). Furthermore, we evaluated the efficiency of an existing feature selection method. In this study, we used the DEA methodology applied in economics to measure the data efficiency.

The contributions of this study are as follows.

- We analyzed the applicability of a methodology used in the game security industry to detect abnormal data in large smart city datasets.
- We applied the proposed approach to the cyber-attack dataset of the industrial control system (ICS).
- We presented a process for finding efficient feature sets that can be applied to the processing of large-scale data using a DEA.

The remainder of this paper is structured as follows. First, some conventional background information on game security, DEA, and anomaly detection in smart cites is presented in Section 2. In Section 3.1, a game-based bot detection methodology is described, and the features are analyzed. The experimental method and the process of applying the DEA are described in Section 3.2 and Section 3.3, respectively. An analysis of the experimental results using the selected dataset is presented in Section 4. In Section 5, we discuss the proposed methodology. We conclude the present study in Section 6.

## 2. Related Studies

### 2.1. Online Game Security

As of 2021, the online game market is worth $23 billion USD [14]. In this study, we focus on massively multiplayer online role-playing games (MMORPGs), which entail many different users accessing a game world simultaneously while playing various roles. Representative MMORPGs include Aion, Maple Story, Lineage, and Black Desert. According to the world’s largest electronic game software distribution network, Steam, their peak concurrent users reached 20 million on 2 January 2021 [15]. We found that even if only the game user’s log data are saved, a vast amount of data must be saved on the servers for these types of games. Various information is included in the log stored in the game server. For example, in MMORPGs, there are logs of the user’s access time, game level, and items collected. These logs are continuously written to the servers.

Furthermore, there are various game items in an MMORPG. Items that are difficult to obtain are occasionally exchanged for real money, rather than game money. Consequently, the money used in these games may have a real-world cash value. Because the value of such game items increases over time, game users have begun to collect them professionally, systematically, and sub-optimally. An automated program that helps collect such items is called a bot, and a group that systematically collects and sells items is called a gold farmer group (GFG).

User retention and churn rate are key factors in the game market. If malicious game users quickly collect items using a bot, this may pose a relative deprivation or disadvantage to normal users, eventually causing game users to leave and later return to the game market. Using the Aion game data as a case study, Kim et al. [16] deployed Pearson and Spearman correlation coefficients to analyze the correlation between user type and activity type. Their experiment revealed a direct correlation between the number of bots and the user churn rate. As the impact of bots on the game industry increases, research is being conducted on bot detection.

Research on abnormal behavior detection in games can be at the client, network, or server level [17,18]. The conventional approach to detecting game bots at the client level is to detect human interaction at the user PC level. These methods are generally based on the CAPTCHA or XIGNCODE technology. A network-level analysis is a method for detecting network information, such as network traffic, based on the IP flow [19]. The server-side analysis method analyzes the game logs stored on the server. These methods are mainly implemented using artificial intelligence. In analyzing a user’s behavior, agents behaving abnormally are defined as bots. Bots and GFGs exhibit different patterns from normal users. These patterns are learned by ML systems to create bot detection models. Unlike other methods, the server-level analysis method is not client-dependent and can be flexibly processed based on internal rules [17]. Game companies have developed various methods to distinguish the patterns of bot activity to enforce the game rules. However, if a malicious actor finds such patterns, this security method can be easily bypassed. Recently, ML and deep learning have been applied to bot detection to prevent pattern-detection bypass attacks.

The online game market collects and processes user logs; therefore, it is naturally involved in the analysis and processing of large-scale data.

### 2.2. DEA Method

The DEA is a method used to measure the efficiency in the field of economics. Efficiency refers to the ratio of the output to the effort or resources invested in a certain objective. Efficiency is measured based on the output and input. The DEA is a relative measure of efficiency, that is, a value expressed relative to the highest level of efficiency.

Charens et al. [20] presented a DEA model characterized by a non-parametric estimation of the relationship between data input and output. The DEA has the advantage of being able to handle a variety of input and output factors. Furthermore, it does not require an assumption regarding the functional relationship. It can also handle the input and output elements at different scales. By using available data, the DEA eliminates the need to create separate data for a performance measurement, and reveals the best practices.

The DEA measures and evaluates the relative performance in the decision-making units (DMUs). It is essential to ensure the DMUs are homogeneous because the DEA compares them. Fitzsimmons et al. [21] stated that the number of DMUs has more influence than double the sum of the input and output variables in discriminating the efficiency measurement results.

Some methods for analyzing the DEA include the Charnes, Cooper, and Rhodes (CCR) [20] and Banker, Charnes, and Cooper (BCC) [22] approaches. The CCR is a model of constant-scale returns with inputs and outputs. By contrast, the BCC is a variable-scale return model with input and output. In the CCR method, the input and output are directly proportional, unlike in the BCC approach. The CCR doubles the output when the input is doubled. For the BCC, doubling the input does not double the output. The DEA model is composed of input- and output-oriented models. This depends on the variable focused upon and whether this variable is an input or output variable. The input-oriented model minimizes the inputs to produce a given output, and the output-based model maximizes the output using a given input. The input and output variables used in this study are variable-scale returns; we used an output-oriented model because it was necessary to maximize the output. Therefore, we used the calculation-oriented variable-scale return-envelopment-calculation-based model. Curi et al. [23] used the variable return of scale (VRS) for the envelopment-output orientation model to estimate the technical efficiency of Italian airports. As a result of their efficiency analysis, they found that the reallocation of traffic at airports close to each other is vital.

The VRS for an envelopment-output orientation model [24] is expressed as a mathematical model, where the set of inputs is I={1,…,m}, the set of outputs is O={1,…,m}, and the set of DMUs is S={1,…,m}. An s×m matrix *X* represents the input, where $x_{i}$ is the column vector of the input connected to $DMU_{i}$ and $x_{ij}$ is the amount the ith DMU uses for input *j*. An s×n matrix *Y* represents the output. Furthermore, $y_{i}$ is the column vector of the output associated with $DMU_{i}$, and $y_{ij}$ represents the amount the ith DMU produces in the output *j*. Vector λ is a column vector of composite weights related to the envelopment of the DEA. Let ϵ be a non-Archimedean element, i.e., a number less than a positive real number. Let $s_{i}^{+}$ and $s^{-}$ be vectors of slack variables for the output and input, each.

(1) $$\begin{array}{c} max\;\;\phi +\epsilon (\sum_{i\in I}S_{i}^{-}+\sum_{i\in O}S_{j}^{+})\\ st:\;\sum_{r\in S}x_{ri}\lambda_{r}+s_{i}^{-}=x_{Oi},\;for\;\;i\in I\\ \phi y_{Oj}-\sum_{r\in S}y_{rj}\lambda_{r}+s_{j}^{-}=0,\;for\;\;j\in O\\ \sum_{r\in S}\lambda_{r}=1\\ \lambda_{r},s_{j}^{+},s_{i}^{-}≧0,i\in S,i\in I,j\in O \end{array}$$

In this study, we calculated these weights by considering the characteristics of our competitors. The DEA assesses the effectiveness of a particular system by measuring the ratio of the weighted sum of the outputs to that of the inputs [20]. The weight used is not a fixed value. In solving the optimization problem, not only are the characteristics of the system considered, the weights of the competitors are also calculated. We measured the efficiency using pyDEA. Along with the DEA method, the experiment conducted in this study is described in Section 3.4.

### 2.3. Anomaly Detection in Smart Cites

Prior research on anomaly detection in large datasets from smart cities was analyzed.

Alrashdi et al. [9] conducted research to detect abnormal behavior in data collected from various Internet of Things (IoT) devices in smart cities. We mainly confined ourselves to the framework of anomaly detection in the IoT (AD-IoT system), using an intelligent anomaly detection technique based on a random forest, which is an ML algorithm for detecting abnormal behavior in IoT networks. The network intrusion detection system (NIDS)-based approach was introduced, and while prior NIDS implementations have the disadvantage of not detecting new attacks, our proposed methodology can reveal new attacks with an accuracy of 99.34%. However, the authors did not verify the performance. In our study, we verified the NIDS-based approach using n-fold cross-validation.

Garcia-Font et al. [25] used ML to detect anomalies in the data from sensor networks and other sources depended on by smart cities. The data were created by reproducing the complex environment of a smart city and evaluated using SVM and an isolation forest algorithm. It was shown that additional considerations are needed to effectively detect attacks on smart cities. It was observed that this study implies a requirement for a multidisciplinary team composed of highly specialized operators in data analysis, network management, and security roles. Therefore, it was assumed that the proposed process was analyzed by experienced experts. In addition, Garcia-Font et al. [26] compared the results with those of an anomaly detection technique that is often used on an actual dataset from the Barcelona Smart City project. Among them, we concluded that one-class SVMs are the most effective technology. In this study, we used an SVM to detect abnormal behavior.

Bawaneh et al. [27] proposed an occupancy-based anomaly detection algorithm to detect abnormal behaviors in road traffic in smart cities. To analyze the time series data, an extended expression sequence was transformed. The modified z-score was used to detect anomalous behavior in the global heat data. We transformed the modified z-score into a sequence and applied the method to our experiment using statistics.

Korzhuk et al. [28] used a random forest classifier to detect attacks in WSNs. In addition, it has been shown that the WSN algorithm developed based on probabilistic classification can reduce the network load of low-power sensor network devices. We applied the random forest algorithm to our method and referenced the probabilistic basis for feature extraction.

As discussed above, research is being conducted to analyze heterogeneous data for detecting abnormal behavior in smart cities. However, in a study using the dataset of an actual smart city, the dataset was not disclosed. In many of the studies that involved simulating a virtual environment, sensor network data were mainly used. Therefore, we applied the ICS sensor dataset in our experiment.

## 3. Proposed Method

### 3.1. Insight from Analysis of Game Bot Detection Methods

In this study, we analyzed a server-side bot detection methodology for detecting abnormal behavior. The features used for bot detection were analyzed based on the characteristics of our experimental data. In this study, the server-side bot detection methodology features used to detect abnormal behavior were analyzed and applied during the experiments. We examined prior studies on the features used in the server-level bot detection methodology. The features determined included a trading network, gameplay style, social network, sequence analysis, self-similarity, character movement, and character behavior. Table 1 describes the criteria used for classifying the features applied in our data-driven security technology research.

**Table 1:** Classification of features used in game bot detection.

Feature Category	Description	Related Works
Trading network	Examining a game character’s possession event log and transaction event log to derive it as a feature	[29,30]
Gameplay style	Investigating gameplay styles such as player information, player action, and combat ability	[31,32]
Social network	Analysis of social network characteristics between players such as part play logs and chat logs	[33,34,35]
Sequence analysis	Characterized by assuming that the player’s actions, such as action sequences and battle sequences, are one sequence	[36,37,38]
Self-similarity	Analyzed based on the assumption that the bots have self-similarity, and the action frequency and action type are used as features	[13,39]
Character movement	Identifying a character’s movement pattern and use movement speed, distance, and location	[40,41]
Character behavior	Observering the character’s behavior and using it as a feature by applying various statistics	[17,42,43,44,45]

We analyzed the logs generated in a smart city’s sensor layer using the hardware-in-the-loop (HIL)-based augmented ICS (HAI 1.0) dataset, which is an infrastructure dataset, as a case study. Furthermore, we investigated the applicability of the proposed approach to such sensor logs and determined how to best apply it. Because the log of the actual sensor layer is a time series log, we explored how to apply the features of the user behavior log detection method. The features used in the referenced game study were analyzed, as shown in Table 2, prior to being applied during this experiment. In this analysis, the server-level analysis method used in the game industry was applied to the time series datasets of the ICS.

A study in which a trade network, such as increases and decreases of a character’s stock, was analyzed was not applicable because our data are time series and not network data [29,30]. In terms of the gameplay style, the combat ability and game player information were analyzed [31,32]. In Chung et al. [31], the standard deviation and similarity were used to differentiate players from bots. Kang et al. [32] statistically analyzed the players’ styles. We matched the similarities and statistics to this approach and applied them to the experimental data. A social network analysis was conducted mainly to analyze the conversations between players and party play logs within the game [33,34,35]. Although Oh et al. [34] used ML for natural language processing, natural language does not exist in our experimental data. In Kang et al. [35], the party player logs related to social interaction were used; however, the interaction data between sensors cannot be known. In the case of a sequence analysis, the action and battle sequences were analyzed to detect any bots [36,37,38].

**Table 2:** Summary and analysis of applicable features.

Author	Feature					
	Standard Deviation	Min	Max	Similarity	Skewness	Kurtosis
Chung et al. [31]				•	
Kang et al. [32]		•	•		•	•
Lee et al. [13]		•	•	•	
Thawonmas et al. [39]		•	•		
Mishima et al. [40]	•				
Chen et al. [41]	•			•	
Yu et al. [42]	•			•	
Han et al. [44]	•			•	
Chen et al. [45]				•	•	•
Park et al. [17]		•	•	•	•	•

Xu et al. [38] calculated the similarity between the game bots and normal users using the Levenshtein distance. However, this method is inapplicable to our data because a distance calculation is impossible. In the case of self-similarity, the behavior patterns of humans and bots are similar, and features such as the max or mean were extracted using statistical features by comparing the difference between the actor and expected human action frequencies [13,39]. Bots were detected by calculating the standard deviation and similarity of the character movement distance [40,41]. In Han et al. [44], through a character behavior analysis, features such as the behavior-related win rate and EXP variance exhibited by the game characters, were extracted. Park et al. [17] normalized these data by counting the actions leading up to a level-up event. We derived the features that have been frequently used in preceding gaming studies, such as the standard deviation, min, max, similarity, skewness, and kurtosis, based on the features of our dataset. The details of the actual application of our experiment are given in Section 3.2.

### 3.2. Feature Extraction

Before applying our methodology, we can consider a statistically based anomaly detection method based on the z-score. For this, we conducted normality tests for each sensor value in the dataset. As a result of testing using a SciPy normaltest, the *p*-value indicating normality was less than 0.001, indicating that the null hypothesis was rejected, and thus the data did not have normality. We therefore conducted an anomaly detection using a machine learning methodology based on the feature extraction.

We applied our approach to the ICS dataset based on the features analyzed in Section 3.1. The dataset is described in Section 4.1. The HAI 1.0 dataset was developed as a time series for anomaly detection research in the ICS.

A standard deviation is the number representing the spread of the data. This is the positive square root of the variance, defined by Equation (2), where σ is the standard deviation, *x* indicates each value in the dataset, $\bar{x}$ is the mean of all values in the dataset, *n* is the number of values in the dataset, and $\tilde{x}$ is the median.

(2) $$\sigma =\sqrt{\frac{\sum (x-\bar{x})}{n}}$$

Min is the minimum value in the time window, and max is the maximum value. Skewness, which is defined by Equation (3), is a measure of the asymmetry, as the data distribution shape tends to be skewed from the center of the mean. Equation (4) provides the kurtosis, which is a measure of whether the distribution of the data is sharper or flatter than the normal distribution.

(3) $$Skewness=\sqrt{\frac{3(\bar{x}-\tilde{x})}{\sigma }}$$

(4) $$Kurtosis=\frac{1}{n}\frac{\sum_{i=1}^{n}{\left(x_{i}-\bar{x}\right)}^{4}}{\sigma^{4}}$$

The similarity is the degree of correlation and indicates whether the same number is repeated in the time series data. Thus, this indicates the extent to which the same state is maintained. We calculated the similarity using the longest consecutive streak. We used the statistical library, SciPy, and the math library, NumPy, to extract six features.

### 3.3. Modeling and Evaluation

Among the algorithms used to detect bots in the game field, we selected the support vector machine (SVM), random forest, decision tree, *k*-nearest neighbor (*k*-NN), and light gradient boosting machine (LightGBM) algorithms in our experiments. The SVM is a supervised learning model for pattern recognition and data analysis. As a binary classifier used for classification and regression analysis that classifies two categories, it has high accuracy because it is not significantly affected by noisy data. A random forest is a supervised learning algorithm and an ensemble model of a decision tree. It creates multiple decision trees and combines them to enable more accurate and stable predictions. *k*-NN is an algorithm used to find the k-nearest neighbors, which are the k elements closest to the input in a specific space and classifies them as more consistent. Although the learning speed is high, depending on the data size, it takes considerable time because all data must be calculated. Decision tree algorithms learn the rules in the target data and create a tree-based classification rule. Although they have a low accuracy compared to other algorithms, their strength is high analysis speed. The LightGBM is a boosting algorithm that uses a leaf-wise method. This algorithm is faster to learn and automatically transforms and optimally segments categorical features.

We used a validation set approach and K-fold cross-validation for verification and evaluation, and we evaluated the experimental results using the F1-scores. The F1-score is the harmonic mean of the precision and recall. We decided to consider abnormal behavior as true and normal behavior as false. The F1-score is shown in Equation (5) and is used as a criterion for a model evaluation. It has a characteristic in that the model can be evaluated without being biased against a specific index by considering both the precision and recall. Precision refers to the ratio of abnormal behavior to the behavior detected as abnormal, and recall is the ratio of accurate detection of abnormal behavior.

(5) $$F1=2\times \frac{Precision\times Recall}{Precision+Recall}$$

### 3.4. Proposed Process Applying DEA Method

**Figure 1:**
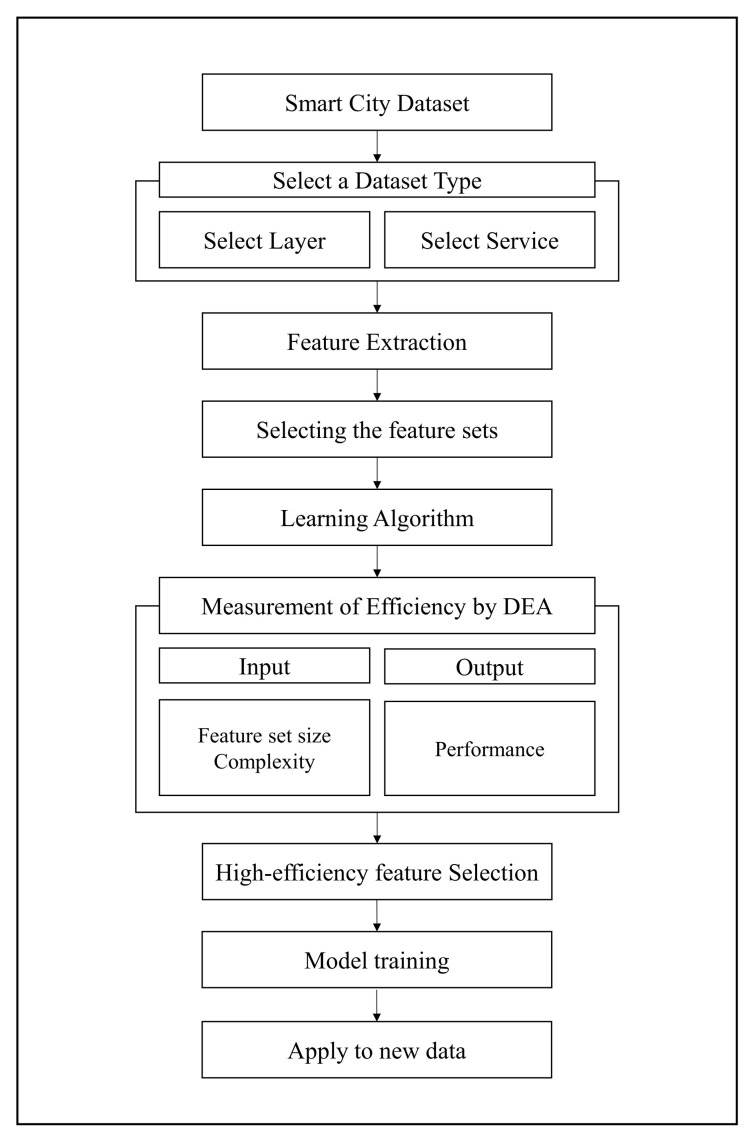
Process measuring efficiency of features by applying DEA.

We implemented the process shown in Figure 1 to determine the most efficient features for detecting abnormal symptoms in smart cities. This process uses the DEA methodology to analyze the efficiency of the proposed approach. The DEA method is described in detail in Section 2.2. We assumed that an experienced expert analyzed the features.

When a smart city dataset is compiled, it is necessary to analyze the layer to which it belongs as well as its service characteristics. The heating control system, which provides heat to homes in a smart city, can be used as a case study. Because it is a heating control system, it belongs to the sensor layer. Therefore, because sensor data are time series data, if administrators wish to analyze the system data, a feature extraction must be applied according to the time series characteristics. In addition, because it is a service for providing heat, if the temperature rises above a specific degree, it can be designated as abnormal behavior and analyzed.

To determine whether the trained model has learned efficient features, we proposed a method for measuring the efficiency of our approach using the DEA method for each feature set. Although it is beneficial to use all of the various features in the experiment, it is more efficient to apply this method after determining the useful feature set. This is because, although more features correspond to greater accuracy, the analysis can consume considerable resources. There may be insufficient system resources to analyze a sizable smart city dataset. Therefore, it is essential to use efficient features.

An experienced expert analyzed and extracted the suitable features. We selected all combinations of the feature sets. Using these feature sets, a model was trained using an algorithm that fits the dataset characteristics. The selected dataset was then evaluated using the previously verified and labeled dataset. We measured the F1-score by applying it to the model trained on the test data. The F1-score from this calculation was used as the performance and output values of the DEA.

We describe the relevant concept in Section 2.2. In our methodology, the DMU is a combination of feature sets to be compared. For example, we set the combination of six features used in our experiment as the DMUs. The number of DMUs was set to $63(=2^{6}-1)$. The input is the feature set size, which is the number of feature types, and the complexity, which is the time required to select a feature per sample.

After checking the efficiency measured using the DEA, the model was retrained using features with high efficiency. Finally, we applied the model to the new data.

The HAI 1.0 data, which were tested for application to a game world approach, were applied in our analysis. The performance was measured using 80% of the data, and the efficient features were selected. The remaining 20% of the data were used to verify the relevance of the methodology. We found it noteworthy that the proposed process also considers the efficiency in the feature selection method, which itself only considers the performance. In addition, an efficiency measurement method applied in economics was applied. The detailed operation method uses the following pseudo-code in Algorithm 1.
**Algorithm 1:** DEA based on the feature selection for anomaly detection in smart city
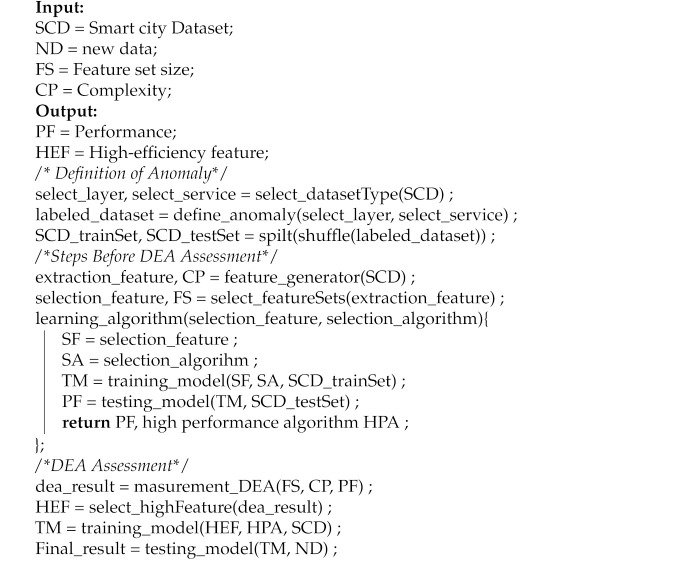


## 4. Experiments

### 4.1. Dataset

We used an ICS dataset, i.e., the HAI 1.0 dataset, for our experiments. Developed by the Affiliated Institute of ETRI in Daejeon, Korea, the HAI 1.0 dataset is made up of time series data and consists of 998,942 time windows. It was created to study a cyber-physical system (CPS) using ML and can be downloaded through GitHub [46]. The data were created in February 2020 and were developed for research on the detection of abnormal behavior in ICSs. Shin et al. [46] designed a simulated control system testbed using industrial control devices, sensors, and actuators, such as those by GE, Emerson, and Siemens, based upon which they developed the ICS security dataset, HAI 1.0. The testbed contains a boiler, turbine, water treatment, and HIL simulation.

We only used data labeled as normal and abnormal. Therefore, we used 990,000 time windows and 980,697 normal and 18,303 abnormal data.

### 4.2. Experimental Setup

**Figure 2:**
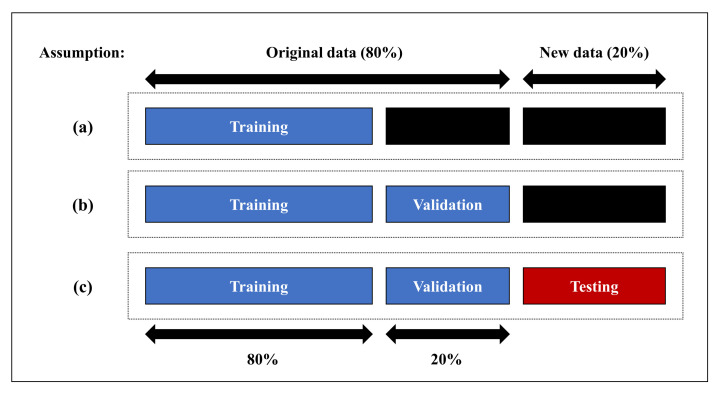
Dataset divided for experimental validation.

As shown in Figure 2, we assumed that 80% of the data were original data and 20% of the data were new. To apply the features analyzed in Section 3.1, the training dataset of the original data was used to train the model in Figure 2a. We used a K-fold cross-validation for verification and evaluation. We selected the model with the best-performing algorithm and time window size based on the evaluation results. This model was used to measure the efficiency of the DEA, as described in Section 3.4. We measured the F1-score of the original data, as shown in Figure 2b, using a validation set approach. The F1-score was used as an input value and for determining the performance of the DEA. Furthermore, to evaluate the proposed process, we measured the F1-score, as shown in Figure 2c using new data.

For the experiment, a Xeon CPU (128 GB of RAM) provided by NIPA was used. To train the five ML algorithms selected above, basic parameters provided by scikit-learn in the Python library were applied as parameters. In the process proposed in Section 3.4, pyDEA was used to measure the efficiency. The package was downloaded from GitHub [47], and the DEA was developed at the University of Auckland, Department of Engineering Science by Kane Harton.

### 4.3. Evaluation Results

Through 10-fold validation, we conducted an experiment to determine the model to use for the experiment. Because they can be considered in advance for use in an efficiency evaluation, random forest, decision tree, *k*-nearest neighbor, and LightGBM algorithms were applied. In addition, because the HAI dataset is made up of time series data in which each ICS sensor information source is recorded in chronological order, it is essential to define an appropriate time window length. To determine an appropriate time window length, we set 30, 60, and 90 s as targets for comparison.

**Figure 3:**
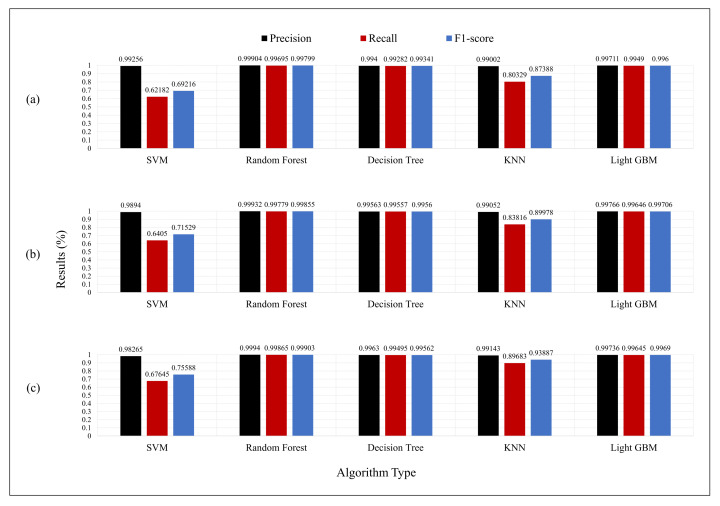
Result of model training. The time window size is (**a**) 30 s, (**b**) 60 s, and (**c**) 90 s.

The experimental results obtained through an evaluation of the model by extracting the game features are shown in Figure 3. Using all game features presented above, we confirmed that the accuracy, precision, and F1-score of all models, except for the SVM and *k*-NN models, were above 90% for all time window sizes during the experiment. We analyzed the results yielded by the model and determined that abnormal behavior in the ICS can be detected using the features designed for game bot detection. Our experimental results showed that three time window sizes of 30, 60, and 90 s have sufficient data to reveal anomalies in the ICS dataset. As a result of experimenting under the three time window conditions, the accuracy, recall, and F1-score all showed a better performance than the other models in a random forest. We confirmed that the optimal rule for anomaly detection was extracted owing to the random forest tree ensemble. Furthermore, between the random forest within a separate time window, we found that the longest (90 s) time window performed the best. Because the 90 s time window has the most significant amount of information in comparison to the 30 and 60 s time windows, it easily captures anomaly signals. For DEA-based feature selection, we used the random forest model, which performed the best, based on the model evaluation results and a time window of 90 s.

We also used a precision-recall curve for determining model performance. the precision-recall-curve is known for giving useful information to compare model performance [48]. Many studies already used it for model selection. In Dionysios et al. [49], they used a precision-recall curve for determining the threshold for classifying potential voice disorders in the Greek population, which showed imbalanced labels. We compared precision recall-curve for 90 s of time window, the random forest showed the best performance as the same as the above experiment results.

### 4.4. Efficiency Analysis Results

We present a methodology for analyzing the efficiency of the features in Section 3.4. The experiments were conducted using HAI 1.0. We used a validation set approach, and the LightGBM algorithm was applied for this efficiency evaluation. The 63 feature sets were permutations of four features. A total of 63 feature combinations were then set as the DMUs. The performance measured for each DMU was used as the output value. The feature set sizes and complexities were used as the input values. The feature size is the number of feature types, and the complexity is the execution time required for selecting the features for each sample.

Table 3 shows the features with the highest efficiency based on the results of the DEA measurement. The efficient feature sets achieved a good performance, with an F1-score of 0.997. To verify this, we retrained the model using the original data and known efficient features. The F1-score of the new data was then evaluated. The F1-score of 0.997 indicated a good performance, as shown in Table 3.

**Figure 4:**
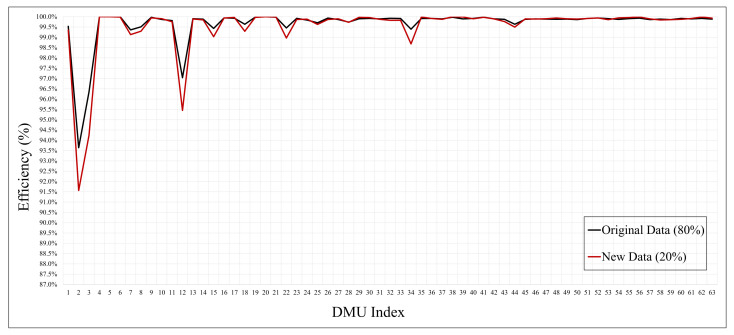
Feature efficiency of the original data (80%) and new data (20%) of each DMU.

The graph in Figure 4 showed that the efficiency was low for a specific feature. As a result of the experiment, there was no significant difference in the overall performance, although among the features, min and max showed an excellent efficiency of 1, which was achieved because of the low complexity and high performance. As the graph in Figure 4 indicates, our proposed process is valid with an efficiency similar to that achieved using either the original or the new data as measured using the DEA method. This implies that the feature set of the original data adjudged as efficient can be applied as new data. The full experimental results are presented in the Appendix A.

## 5. Discussion

In this study, methodologies for game bot detection were derived from online game research and applied to the HAI ICS dataset representing normal and abnormal smart city data.

- The performance of the *k*-NN, random forest, decision tree, and LightGBM models were evaluated, and the random forest model showed the best performance.
- We tested three different conditions about the time window for extracting features. Moreover, we found that the 90 s for a time window is the best condition with random forest showed an F1-score of 0.99903.
- In addition, a feature selection method based on the DEA, which was previously used to measure the efficiency of the data, was proposed.
- The min, max, and similarity features showed the best efficiency through the experimental result. We tried to find out why these three features are better than others. We found that when criminals attack ICS, the ICS sensors showed abnormal values like over the maximum limit of sensors; under the minimum limitation of sensors, the values do not fluctuate. The feature sets we generated are well reflected this attacked situation and showed relevant results for proposed methods.
- This approach considers the performance of the existing feature selection algorithms, as well as the complexity and size of the feature set.
- In addition to preventing overfitting, which is commonly discussed in data-based abnormal behavior detection, this methodology makes it easy to apply practical feature datasets; furthermore, it guarantees a high performance.

We analyzed the game bot detection approach and utilized it in the cybersecurity field for smart cities. We experimented on an ICS dataset that can be used in smart cities. However, this dataset was not actually the data from a smart city, but was rather the linked infrastructure dataset. Considerable resources are required to analyze the large-scale data of smart cities and achieve an effective model. Therefore, our process makes it possible to analyze the efficiency through a model trained using some smart city data and select the feature sets with the highest efficiency. An anomaly detection model can be created by retraining the dataset labeled using the selected feature sets.

## 6. Conclusions

We presented a method for analyzing the massive data generated by smart cities. Through this approach, a process model was created based on the DEA technique. In addition, to analyze smart city data, an approach used in game security was analyzed. Online game security features were analyzed and related to actual infrastructure data. The results of the experiment showed that the F1-score value was 0.99865, indicating a high performance. Furthermore, our study confirmed that the features used in the actual game security model are similar to the sensor layer data.

The HAI 1.0 dataset used in our experiment is sensor data from the ICS. However, it cannot be interpreted in terms of user behavior. Unfortunately, a smart city user dataset is not publicly available. In future studies, similar to game bot detection, a dataset that can be used in studies on screening out abnormal actors in a smart city may be applied.

Furthermore, feature extraction is not an automated process in the deep learning method. Hence, when incorporated into the DEA method, it is ineffective at selecting the key features. However, deep learning is commonly known to outperform the previously proposed ML-based methodology; therefore, it cannot be overlooked. In future studies, we can consider measuring the efficiency by analyzing the training time of the deep learning model.

This approach is also expected to be implementable for various smart city datasets through the proposed process. In this study, we performed anomaly detection for sensor data from the infrastructure of a smart city. In a future study, we can study anomaly detection methods for healthcare data, including sensitive information of smart city users. By detecting the presence of abnormal behaviors, we will contribute to the safe construction and operation of smart cities.

## Figures and Tables

**Table 3:** Results of feature efficiency using DEA method.

	Original Data (80%)				New Data (20%)			
DMU (Feature List)	Feature Set Size	Complexity	Performance	Efficiency	Feature Set Size	Complexity	Performance	Efficiency
(’max’)	1	0.002334118	0.997300786	1	1	0.002334118	0.997579382	1
(’min’)	1	0.001845837	0.996949073	1	1	0.001845837	0.997436628	1
(’similar’)	1	0.141670704	0.997534922	1	1	0.141670704	0.997435897	0.999856121
(’max’, ’similar’)	2	0.144004822	0.997653684	1	2	0.144004822	0.998006266	1

## Abbreviations

The following abbreviations are used in this manuscript:

BCC	Banker, Charnes and Cooper
CCR	Charnes, Cooper, and Rhodes
CPS	Cyber-Physical System
DEA	Data Envelopment Analysis
DMU	Decision Making Unit
EXP	Experience point
GBM	Gradient Boosting Machine
GFG	Gold Farming Guide
HIL	Hardware-in-the-Loop
ICS	Industrial Control System
IoT	Internet of Things
IIoT	Industrial Internet of Things
KNN	*k*-Nearest Neighbors
MMORPG	Massively Multiplayer Online Role-Playing Game
NIDS	Network Intrusion Detection System
STD	Standard Deviation
SVM	Support Vector Machine
VRS	Variable Return to Scale
WSN	Wireless Sensor Networks

## Appendix A

**Table A1:** This appendix shows the results of measuring the DEA method using pyDEA, for which we applied the output-oriented VRS model. The detailed values are shown in Figure 3.

		Original Data (80%)				New Data (20%)			
Index	DMU (Feature List)	Feature Sets Size	Complexity	Performance	Efficiency	Feature Sets Size	Complexity	Performance	Efficiency
1	(’std’)	1	0.021793127	0.992683502	0.99533754	1	0.021793127	0.991109837	0.993514733
2	(’kurt’)	1	0.014263153	0.933899788	0.936408587	1	0.014263153	0.913419913	0.915636294
3	(’skew’)	1	0.00265193	0.960925137	0.963525362	1	0.00265193	0.940165787	0.942447111
4	(’max’)	1	0.002334118	0.997300786	1	1	0.002334118	0.997579382	1
5	(’min’)	1	0.001845837	0.996949073	1	1	0.001845837	0.997436628	1
6	(’similar’)	1	0.141670704	0.997534922	1	1	0.141670704	0.997435897	0.999856121
7	(’std’, ’kurt’)	2	0.03605628	0.991016548	0.99361503	2	0.03605628	0.98908046	0.991330026
8	(’std’, ’skew’)	2	0.024445057	0.992445703	0.995076857	2	0.024445057	0.990675656	0.992958337
9	(’std’, ’max’)	2	0.024127245	0.997063315	0.999707486	2	0.024127245	0.997150997	0.999449403
10	(’std’, ’min’)	2	0.023638964	0.996002822	0.998645338	2	0.023638964	0.996725046	0.999023754
11	(’std’, ’similar’)	2	0.163463831	0.995765702	0.998107588	2	0.163463831	0.99557459	0.997563451
12	(’kurt’, ’skew’)	2	0.016915083	0.967819985	0.970404035	2	0.016915083	0.952309985	0.954522628
13	(’kurt’, ’max’)	2	0.016597271	0.996356799	0.999017866	2	0.016597271	0.996578272	0.998894623
14	(’kurt’, ’min’)	2	0.01610899	0.996121753	0.998783382	2	0.01610899	0.996151105	0.998467751
15	(’kurt’, ’similar’)	2	0.155933857	0.991967871	0.994300767	2	0.155933857	0.988340291	0.990314722
16	(’skew’, ’max’)	2	0.004986048	0.99670898	0.99939996	2	0.004986048	0.997007268	0.999354317
17	(’skew’, ’min’)	2	0.004497766	0.996591043	0.999282915	2	0.004497766	0.997293062	0.999644526
18	(’skew’, ’similar’)	2	0.144322634	0.993986558	0.996324261	2	0.144322634	0.990954774	0.992934378
19	(’max’, ’min’)	2	0.004179955	0.997065383	0.999759358	2	0.004179955	0.997295374	0.999656918
20	(’max’, ’similar’)	2	0.144004822	0.997653684	1	2	0.144004822	0.998006266	1
21	(’min’, ’similar’)	2	0.143516541	0.99741784	0.999764855	2	0.143516541	0.997864769	0.99985942
22	(’std’, ’kurt’, ’skew’)	3	0.03870821	0.991969769	0.994564209	3	0.03870821	0.987487416	0.989676976
23	(’std’, ’kurt’, ’max’)	3	0.038390398	0.996591844	0.999199142	3	0.038390398	0.996437224	0.998647232
24	(’std’, ’kurt’, ’min’)	3	0.037902117	0.995765702	0.998372055	3	0.037902117	0.996580222	0.998791562
25	(’std’, ’kurt’, ’similar’)	3	0.177726984	0.994701519	0.997040882	3	0.177726984	0.994280812	0.996267087
26	(’std’, ’skew’, ’max’)	3	0.026779175	0.99670898	0.999345529	3	0.026779175	0.996435192	0.998669373
27	(’std’, ’skew’, ’min’)	3	0.026290894	0.996000941	0.998636861	3	0.026290894	0.996722246	0.998958087
28	(’std’, ’skew’, ’similar’)	3	0.166115761	0.995055333	0.997395501	3	0.166115761	0.995289079	0.997277433
29	(’std’, ’max’, ’min’)	3	0.025973082	0.996358511	0.998996209	3	0.025973082	0.997438087	0.999676205
30	(’std’, ’max’, ’similar’)	3	0.165797949	0.99682987	0.999174282	3	0.165797949	0.997579382	0.999572283
31	(’std’, ’min’, ’similar’)	3	0.165309668	0.996592645	0.998936432	3	0.165309668	0.996724113	0.998715253
32	(’kurt’, ’skew’, ’max’)	3	0.019249201	0.996590241	0.99924527	3	0.019249201	0.996004566	0.998253356
33	(’kurt’, ’skew’, ’min’)	3	0.01876092	0.996472248	0.999128261	3	0.01876092	0.996004566	0.998254452
34	(’kurt’, ’skew’, ’similar’)	3	0.158585787	0.991608557	0.99394064	3	0.158585787	0.984833165	0.986800556
35	(’kurt’, ’max’, ’min’)	3	0.018443108	0.996475564	0.999132353	3	0.018443108	0.997580071	0.999834128
36	(’kurt’, ’max’, ’similar’)	3	0.158267975	0.996826889	0.999171287	3	0.158267975	0.997150997	0.999143035
37	(’kurt’, ’min’, ’similar’)	3	0.157779694	0.996593445	0.998937231	3	0.157779694	0.996724113	0.998715253
38	(’skew’, ’max’, ’min’)	3	0.006831884	0.997062625	0.999749963	3	0.006831884	0.997721447	1
39	(’skew’, ’max’, ’similar’)	3	0.146656752	0.996593445	0.998937231	3	0.146656752	0.997862334	0.999855821
40	(’skew’, ’min’, ’similar’)	3	0.14616847	0.996826889	0.999171287	3	0.14616847	0.997008121	0.998999901
41	(’max’, ’min’, ’similar’)	3	0.145850658	0.997419052	0.999764855	3	0.145850658	0.997723392	0.99971658
42	(’std’, ’kurt’, ’skew’, ’max’)	4	0.041042328	0.996355085	0.998955193	4	0.041042328	0.996721311	0.998926454
43	(’std’, ’kurt’, ’skew’, ’min’)	4	0.040554047	0.996119017	0.998719741	4	0.040554047	0.995435093	0.99763839
44	(’std’, ’kurt’, ’skew’, ’similar’)	4	0.180378914	0.993992225	0.996329919	4	0.180378914	0.992986976	0.994970721
45	(’std’, ’kurt’, ’max’, ’min’)	4	0.040236235	0.996121753	0.998723232	4	0.040236235	0.996724113	0.998930944
46	(’std’, ’kurt’, ’max’, ’similar’)	4	0.180061102	0.996592645	0.998936432	4	0.180061102	0.996866097	0.998857507
47	(’std’, ’kurt’, ’min’, ’similar’)	4	0.179572821	0.996474736	0.998818298	4	0.179572821	0.997008121	0.998999901
48	(’std’, ’skew’, ’max’, ’min’)	4	0.028625011	0.996119017	0.998749466	4	0.028625011	0.997150997	0.999382881
49	(’std’, ’skew’, ’max’, ’similar’)	4	0.168449879	0.996475564	0.998819096	4	0.168449879	0.997007268	0.998999003
50	(’std’, ’skew’, ’min’, ’similar’)	4	0.167961597	0.996239718	0.998582712	4	0.167961597	0.996865204	0.998856609
51	(’std’, ’max’, ’min’, ’similar’)	4	0.167643785	0.996829125	0.999173484	4	0.167643785	0.99715343	0.999145431
52	(’kurt’, ’skew’, ’max’, ’min’)	4	0.021095037	0.99670898	0.99935981	4	0.021095037	0.997150997	0.999398562
53	(’kurt’, ’skew’, ’max’, ’similar’)	4	0.160919905	0.996708206	0.999052299	4	0.160919905	0.996578272	0.99856915
54	(’kurt’, ’skew’, ’min’, ’similar’)	4	0.160431623	0.996355942	0.998699194	4	0.160431623	0.997435167	0.999427728
55	(’kurt’, ’max’, ’min’, ’similar’)	4	0.160113811	0.996710526	0.999054595	4	0.160113811	0.997580071	0.999572982
56	(’skew’, ’max’, ’min’, ’similar’)	4	0.148502588	0.996946206	0.999290903	4	0.148502588	0.997864769	0.99985822
57	(’std’, ’kurt’, ’skew’, ’max’, ’min’)	5	0.042888165	0.996002822	0.99859737	5	0.042888165	0.996722246	0.99892356
58	(’std’, ’kurt’, ’skew’, ’max’, ’similar’)	5	0.182713032	0.996473078	0.998816602	5	0.182713032	0.996436208	0.998426779
59	(’std’, ’kurt’, ’skew’, ’min’, ’similar’)	5	0.182224751	0.996237065	0.998580019	5	0.182224751	0.996580222	0.998571145
60	(’std’, ’kurt’, ’max’, ’min’, ’similar’)	5	0.181906939	0.99682838	0.999172785	5	0.181906939	0.996724113	0.998715253
61	(’std’, ’skew’, ’max’, ’min’, ’similar’)	5	0.170295715	0.996592645	0.998936432	5	0.170295715	0.997151809	0.999143834
62	(’kurt’, ’skew’, ’max’, ’min’, ’similar’)	5	0.162765741	0.996827635	0.999171986	5	0.162765741	0.997864161	0.99985762
63	(’std’, ’kurt’, ’skew’, ’max’, ’min’, ’similar’)	6	0.184558868	0.996473907	0.9988174	6	0.184558868	0.997294603	0.999286909

## References

[1] Neirotti P., De Marco A., Cagliano A.C., Mangano G., Scorrano F. (2014). Current trends in Smart City initiatives: Some stylised facts. Cities.

[2] Nam T., Pardo T.A. Conceptualizing smart city with dimensions of technology, people, and institutions. Proceedings of the 12th Annual International Digital Government Research Conference: Digital Government Innovation in Challenging Times.

[3] Washburn D., Sindhu U., Balaouras S., Dines R.A., Hayes N., Nelson L.E. (2009). Helping CIOs understand “smart city” initiatives. Growth.

[4] Chourabi H., Nam T., Walker S., Gil-Garcia J.R., Mellouli S., Nahon K., Pardo T.A., Scholl H.J. Understanding smart cities: An integrative framework. Proceedings of the 2012 45th Hawaii International Conference on System Sciences.

[5] Khatoun R., Zeadally S. (2017). Cybersecurity and privacy solutions in smart cities. IEEE Commun. Mag..

[6] Liu X., Nielsen P.S. (2018). Scalable prediction-based online anomaly detection for smart meter data. Inf. Syst..

[7] U.S. Cybersecurity Firm FireEye Discloses Breach, Theft of Hacking Tools. https://uk.reuters.com/article/fireeye-cyber/u-s-cybersecurity-firm-fireeye-discloses-breach-theft-of-hacking-tools-idUKKBN28I34H.

[8] Difallah D.E., Cudre-Mauroux P., McKenna S.A. (2013). Scalable anomaly detection for smart city infrastructure networks. IEEE Internet Comput..

[9] Alrashdi I., Alqazzaz A., Aloufi E., Alharthi R., Zohdy M., Ming H. Ad-iot: Anomaly detection of iot cyberattacks in smart city using machine learning. Proceedings of the 2019 IEEE 9th Annual Computing and Communication Workshop and Conference (CCWC).

[10] Gaur A., Scotney B., Parr G., McClean S. (2015). Smart city architecture and its applications based on IoT. Procedia Comput. Sci..

[11] Falco G., Viswanathan A., Caldera C., Shrobe H. (2018). A master attack methodology for an AI-based automated attack planner for smart cities. IEEE Access.

[12] Liagkou V., Kavvadas V., Chronopoulos S.K., Tafiadis D., Christofilakis V., Peppas K.P. (2019). Attack detection for healthcare monitoring systems using mechanical learning ual private networks over optical transport layer architecture. Computation.

[13] Lee E., Woo J., Kim H., Mohaisen A., Kim H.K. You are a Game Bot!: Uncovering Game Bots in MMORPGs via Self-similarity in the Wild. Proceedings of the Ndss Symposium.

[14] Online Games-Worldwide. https://www.statista.com/outlook/212/100/online-games/worldwide.

[15] Si-young O. (2021). Steam Co-Existence Surpassed 25 Million ’New Record’. http://it.chosun.com/site/data/html_dir/2021/01/04/2021010400549.html.

[16] Kim Y.H., Yang S.I., Kim H.K. (2018). Correlation Analysis between Game Bots and Churn using Access Record. J. Korea Game Soc..

[17] Park S., Lee K. (2020). The Gravy Value: A Set of Features for Pinpointing BOT Detection Method. Proceedings of the International Conference on Information Security Applications.

[18] Woo J., Kim H.K. Survey and research direction on online game security. Proceedings of the Workshop at SIGGRAPH Asia.

[19] Melnikov N., Schönwälder J. (2010). Cybermetrics: User identification through network flow analysis. Proceedings of the IFIP International Conference on Autonomous Infrastructure, Management and Security.

[20] Charnes A., Cooper W.W., Rhodes E. (1978). Measuring the efficiency of decision making units. Eur. J. Oper. Res..

[21] Fitzsimmons J.A., Fitzsimmons M.J. (1994). Service Management for Competitive Advantage.

[22] Banker R.D., Charnes A., Cooper W.W. (1984). Some models for estimating technical and scale inefficiencies in data envelopment analysis. Manag. Sci..

[23] Curi C., Gitto S., Mancuso P. (2011). New evidence on the efficiency of Italian airports: A bootstrapped DEA analysis. Socio-Econ. Plan. Sci..

[24] Cooper W.W., Seiford L.M., Zhu J. (2004). Handbook on Data Envelopment Analysis.

[25] Garcia-Font V., Garrigues C., Rifà-Pous H. (2018). Difficulties and challenges of anomaly detection in smart cities: A laboratory analysis. Sensors.

[26] Garcia-Font V., Garrigues C., Rifà-Pous H. (2016). A comparative study of anomaly detection techniques for smart city wireless sensor networks. Sensors.

[27] Bawaneh M., Simon V. Anomaly detection in smart city traffic based on time series analysis. Proceedings of the 2019 International Conference on Software, Telecommunications and Computer Networks (SoftCOM).

[28] Korzhuk V., Groznykh A., Menshikov A., Strecker M. (2019). Identification of attacks against wireless sensor networks based on behaviour analysis. J. Wirel. Mob. Netw. Ubiquitous Comput. Dependable Appl..

[29] Fujita A., Itsuki H., Matsubara H. Detecting real money traders in MMORPG by using trading network. Proceedings of the AAAI Conference on Artificial Intelligence and Interactive Digital Entertainment.

[30] Kwon H., Mohaisen A., Woo J., Kim Y., Lee E., Kim H.K. (2016). Crime scene reconstruction: Online gold farming network analysis. IEEE Trans. Inf. Forensics Secur..

[31] Chung Y., Park C., Kim N., Cho H., Yoon T., Lee H., Lee J.H. (2013). Game bot detection approach based on behavior analysis and consideration of various play styles. ETRI J..

[32] Kang A.R., Jeong S.H., Mohaisen A., Kim H.K. (2016). Multimodal game bot detection using user behavioral characteristics. SpringerPlus.

[33] Oh J., Borbora Z.H., Sharma D., Srivastava J. Bot detection based on social interactions in MMORPGs. Proceedings of the 2013 International Conference on Social Computing.

[34] Kang A.R., Kim H.K., Woo J. (2012). Chatting pattern based game BOT detection: Do they talk like us?. KSII Trans. Internet Inf. Syst..

[35] Kang A.R., Woo J., Park J., Kim H.K. (2013). Online game bot detection based on party-play log analysis. Comput. Math. Appl..

[36] Lee J., Lim J., Cho W., Kim H.K. I know what the BOTs did yesterday: Full action sequence analysis using Naïve Bayesian algorithm. Proceedings of the 2013 12th Annual Workshop on Network and Systems Support for Games (NetGames).

[37] Xu J., Luo Y., Tao J., Fan C., Zhao Z., Lu J. (2020). NGUARD+ An Attention-based Game Bot Detection Framework via Player Behavior Sequences. ACM Trans. Knowl. Discov. Data TKDD.

[38] Platzer C. Sequence-based bot detection in massive multiplayer online games. Proceedings of the 2011 8th International Conference on Information, Communications & Signal Processing.

[39] Thawonmas R., Kashifuji Y., Chen K.T. Detection of MMORPG bots based on behavior analysis. Proceedings of the 2008 International Conference on Advances in Computer Entertainment Technology.

[40] Mishima Y., Fukuda K., Esaki H. An analysis of players and bots behaviors in MMORPG. Proceedings of the 2013 IEEE 27th International Conference on Advanced Information Networking and Applications (AINA).

[41] Chen K.T., Liao A., Pao H.K.K., Chu H.H. (2008). Game bot detection based on avatar trajectory. Proceedings of the International Conference on Entertainment Computing.

[42] Yu S.Y., Hammerla N., Yan J., Andras P. A statistical aimbot detection method for online FPS games. Proceedings of the 2012 International Joint Conference on Neural Networks (IJCNN).

[43] Yu S.Y., Hammerla N., Yan J., Andras P. (2012). Aimbot detection in online fps games using a heuristic method based on distribution comparison matrix. Proceedings of the International Conference on Neural Information Processing.

[44] Han M.L., Park J.K., Kim H.K. (2015). Online game bot detection in fps game. Proceedings of the 18th Asia Pacific Symposium on Intelligent and Evolutionary Systems.

[45] Chen K.T., Hong L.W. User identification based on game-play activity patterns. Proceedings of the 6th ACM SIGCOMM Workshop on Network and System Support for Games.

[46] Shin H.K., Lee W., Yun J.H., Kim H. {HAI} 1.0: HIL-based Augmented {ICS} Security Dataset. Proceedings of the 13th {USENIX} Workshop on Cyber Security Experimentation and Test ({CSET} 20).

[47] Raith A., Rouse P., Seiford L.M. (2019). Benchmarking Using Data Envelopment Analysis: Application to Stores of a Post and Banking Business. Multiple Criteria Decision Making and Aiding.

[48] Saito T., Rehmsmeier M. (2015). The precision-recall plot is more informative than the ROC plot when evaluating binary classifiers on imbalanced datasets. PLoS ONE.

[49] Tafiadis D., Chronopoulos S.K., Helidoni M.E., Kosma E.I., Voniati L., Papadopoulos P., Murry T., Ziavra N., Velegrakis G.A. (2019). Checking for voice disorders without clinical intervention: The Greek and global VHI thresholds for voice disordered patients. Sci. Rep..

